# Incidence and predictors of in-stent restenosis following intervention for pulmonary vein stenosis due to fibrosing mediastinitis

**DOI:** 10.1186/s13023-024-03391-8

**Published:** 2024-10-14

**Authors:** Mengfei Jia, Hongling Su, Kaiyu Jiang, Aqian Wang, Zhaoxia Guo, Hai Zhu, Fu Zhang, Xuechun Sun, Yiwei Shi, Xin Pan, Yunshan Cao

**Affiliations:** 1 The First Clinical Medical College of Gansu University of Chinese Medicine (Gansu Provincial Hospital), Lanzhou, 730000 China; 2 Heart, Lung and Vessels Center, Sichuan Provincial People′s Hospital, University of Electronic Science and Technology of China, Chengdu, 610072 Sichuan China; 3 NHC Key Laboratory of Pneumoconiosis, No.85 Jiefang South Road, Taiyuan, 030000 China; 4 Shanxi Province Key Laboratory of Respiratory, Department of Pulmonary and Critical Care Medicine, The First Hospital of Shanxi Medical University, Taiyuan, 030000 China; 5 Department of Cardiology, Shanghai Chest Hospital, Shanghai Jiao Tong University, Shanghai, 200030 China

**Keywords:** Fibrosing mediastinitis, In-stent restenosis, Predictor, Pulmonary vein, Stenting

## Abstract

**Background:**

Fibrosing mediastinitis (FM) is a rare yet fatal condition, caused by different triggers and frequently culminating in the obstruction of the pulmonary vasculature and airways, often leading to pulmonary hypertension and right heart failure. Percutaneous transluminal pulmonary venoplasty (PTPV) is an emerging treatment for pulmonary vein stenosis (PVS) caused by FM. Our previous study showed as high as 24% of in-stent restenosis (ISR) in FM. However, the predictors of ISR are elusive.

**Objectives:**

We sought to identify the predictors of ISR in patients with PVS caused by extraluminal compression due to FM.

**Methods:**

We retrospectively enrolled patients with PVS-FM who underwent PTPV between July 1, 2018, and December 31, 2022. According to ISR status, patients were divided into two groups: the ISR group and the non-ISR group. Baseline characteristics (demographics and lesions) and procedure-related information were abstracted from patient records and analyzed. Univariate and multivariate analyses were performed to determine the predictors of ISR.

**Results:**

A total of 142 stents were implanted in 134 PVs of 65 patients with PVS-FM. Over a median follow-up of 6.6 (3.4–15.7) months, 61 of 134 PVs suffered from ISR. Multivariate analysis demonstrated a significantly lower risk of ISR in PVs with a larger reference vessel diameter (RVD) (odds ratio (OR): 0.79; 95% confidence interval [CI]: 0.64 to 0.98; *P* = 0.032), and stenosis of the corresponding pulmonary artery (Cor-PA) independently increased the risk of restenosis (OR: 3.41; 95% CI: 1.31 to 8.86; *P* = 0.012). The cumulative ISR was 6.3%, 21.4%, and 39.2% at the 3-, 6-, and 12-month follow-up, respectively.

**Conclusion:**

ISR is very high in PVS-FM, which is independently associated with RVD and Cor-PA stenosis.

**Trail Registration:**

Chinese Clinical Trials Register; No.: ChiCTR2000033153. URL: http://www.chictr.org.cn.

**Graphical Abstract:**

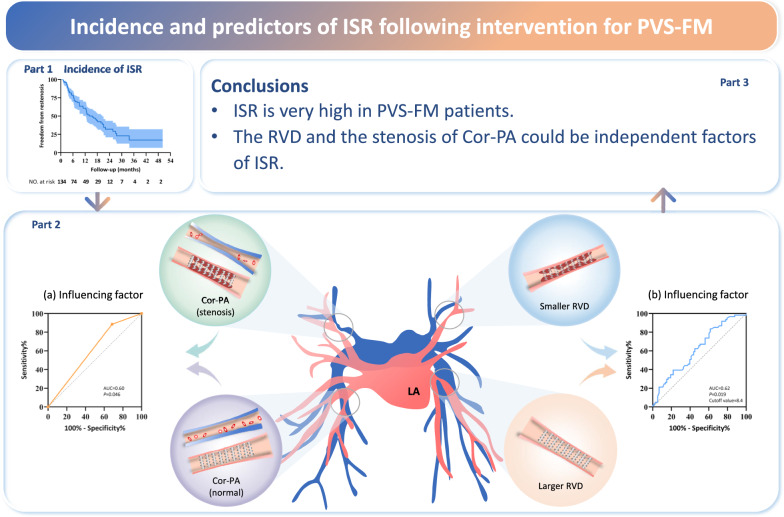

**Supplementary Information:**

The online version contains supplementary material available at 10.1186/s13023-024-03391-8.

## Introduction

Fibrosing mediastinitis (FM) is characterized by benign proliferative fibrous tissue in the mediastinum, often compressing the pulmonary artery (PA), pulmonary vein (PV), bronchi, and superior vena cava, presenting with cough, dyspnea, hemoptysis, pleural effusion, superior vena cava syndrome, pulmonary hypertension, and right heart failure [1]. The most common etiological factors for FM are infection with *Histoplasma capsulatum* in the United States and *Mycobacterium tuberculosis* in China [2]. Pulmonary vein stenosis (PVS) caused by FM (PVS-FM) is a kind of typical extraluminal compressing stenosis that is rare but fatal. Percutaneous transluminal pulmonary venoplasty (PTPV) is an emerging alternative for PVS-FM [3–5].

The first balloon angioplasty (BA) reported by Massumi et al. [6] in 1981 was performed on a female patient with PVS-FM. However, early reports showed that BA was unsuccessful in the treatment of PVS, including a modified BA technique [7, 8]. The first report on endovascular stenting of PVS-FM was in 2001, which brought a new therapeutic modality for FM [9]. In the early application of PV interventions, transcatheter angioplasty was mainly used to correct congenital or postoperative PVS in children [10]. Since the first report of PVS after pulmonary vein isolation (PVI) in 1998, catheter-based intervention has become increasingly common in the treatment of PVS caused by PVI (PVS-PVI) [11]. Nevertheless, detailed information about interventional treatment for PVS-FM, including hemodynamic changes, procedure-related complications, comprehensive follow-up data, incidence, and predictors of in-stent restenosis (ISR), is scarce [12].

The pathogenesis of PVS-FM is different from that of PVS-PVI and congenital PVS. PVS-FM is attributed to extraluminal proliferative fibrous tissue compression [2], while other PVS are attributed to intimal hyperplasia [13, 14]. Hence, even though PTPV has been successfully used in PVS-PVI, PTPV in PVS-FM might differ. Our preliminary data showed that patients with PVS-FM who underwent interventions demonstrated clinical improvement, both in terms of hemodynamics and exercise capacity, but also a high prevalence of restenosis during a very short-term follow-up period [15]. Therefore, identifying the influencing factors associated with ISR is critical to guide intervention and optimize postintervention surveillance strategies. Against this background, we sought to identify the predictors associated with ISR following PVS-FM intervention.

## Methods

### Study population

From July 1, 2018, to December 31, 2022, we identified 144 patients with FM according to history, symptoms, signs, and findings in enhanced computed tomography (CT) with contrast in our center. Patients with PVS caused by tumors and other diseases were excluded. Repeat CT imaging had been routinely performed to evaluate ISR during follow-up. Patients with multiple pulmonary veins undergoing interventional therapy who had both ISR and non-ISR pulmonary veins were present in both groups.

### Data collection

Patient clinical data at baseline and follow-up periods were collected. The procedure-related parameters collected included minimal lumen diameter (MLD), lesion length, and reference vessel diameter (RVD) (taken as the mean diameter of the normal-appearing proximal and distal segment; if the PV diameter at both ends of the stenotic site was greatly different, the diameter of the distal PV served as the reference diameter)**.** Furthermore, the maximal balloon diameter (using the actual measured maximal balloon size), maximal balloon inflation pressure, stent diameter, stent length, maximal stent inflation pressure, final lumen diameter (FLD), balloon-to-vessel ratio (calculated as the largest diameter of the inflated balloon divided by RVD), vessel-to-stent ratio (calculated as the FLD divided by stent diameter), pressure gradients (Pd) and diameter stenosis (%) (calculated as [1-(MLD/RVD)] × 100%) were also included. In addition, information on cases where PA narrowing occurred in series with stenotic PV, accompanied by pleural effusion, and where postoperative anticoagulants were administered, was collected. The pulmonary venous flow grade (PVFG) was assigned using grades 0–3 [15].

### Percutaneous intervention

The procedural approach has previously been described in detail [15]. Informed consent was obtained from the patient for all procedures and operations. The patient was positioned supine, and local anesthesia was administered. Successful femoral venous puncture followed by insertion of 8.5F vascular sheath. Swan-Ganz catheter (*Edward Life Sciences*) was advanced via sheath to inferior vena cava, the right atrium, right ventricle, and pulmonary artery for hemodynamic assessment [16]. The interatrial septum was successfully punctured, and a JR4.0-guiding catheter was advanced through the SWARTZ sheath to the ostium of the target vessel. Subsequently, a Runthrough guidewire was navigated across the lesion to the distal end of the target vessel. Following this, the guiding catheter was maneuvered over the guidewire to the distal end of the stenosis, where pressure measurements and selective pulmonary venography were performed. Upon completion of these procedures, the guiding catheter was retracted to the ostium of the target vessel.

Percutaneous pulmonary vein angioplasty is performed when the angiography shows a narrowing of > 70% [17] or when the Pd between the two ends of the PV narrowing is > 5 mm Hg (1 mm Hg = 0.133 kPa). The normal diameter of a stenotic vessel is predicted by averaging the normal PV diameters at both ends of the stenosis [(proximal PV diameter + distal PV diameter) / 2], or by using the distal PV diameter as the reference diameter when the difference in PV diameter between the proximal and distal ends of the stenosis is significant. According to the predicted diameter and stenosis degree, balloons with corresponding diameters (*Sterling™ or MUSTANGTM, Boston Scientific, USA*) were selected for stepwise pre-dilating. When the predicted diameter of the stenosis PV was < 6 mm, further intervention was abandoned in the presence of elastic recoil of the stenotic vessel or when the target vessel could not be effectively expanded. When the predicted diameter of stenosis PV ≥ 6 mm, the bare metal stent (*Express™ Vascular LD, Boston Scientific, USA*) was implanted when elastic recoil of stenosis vessels existed. The appropriate stent was selected according to the predicted diameter. Acute procedure success was defined as a > 50% increase in the diameter of the previously treated PV and/or > 50% reduction in the Pd across the stenosis. Intraoperative heparin is administered intravenously to adjust the activated clotting time (ACT) to 250-300 s.

After stenting, repeated hemodynamic measurements and angiography assessed the relief of Pd and anatomical obstruction. All patients were anticoagulated with aspirin and rivaroxaban for 3–6 months and followed with rivaroxaban for 3–6 months.

### Outcomes

The primary endpoint of the study was the incidence of ISR following PTPV during the follow-up period, and the secondary endpoints were the World Health Organization functional class (WHO-FC) for pulmonary hypertension and 6-min walking distance (6MWD). We also analyzed the demographic, clinical, and procedural variables associated with ISR.

ISR was defined as stenosis > 50% of the vessel size as confirmed by repeated CT angiography or selected PV angiography or an increase in Pd (≥ 5 mm Hg) across the stenotic site compared to the last measurement.

### Statistical analysis

Categorical data are expressed as counts and proportions (%). Continuous data are reported as the mean ± SD or as the median (interquartile range). The Kolmogorov‒Smirnov test was used to determine the normality of the data distribution. For continuous variables, *t-*tests or Wilcoxon rank sum tests were used as appropriate. For categorical variables, χ^2^ tests or Fisher exact tests were used. A binary logistic analysis was used to construct an optimal model in multiple variables analysis. Receiver operating characteristic (ROC) analyses were used to determine the predictive power of variables for ISR. A 95% confidence interval (CI) is provided for all estimates. A *P* value < 0.05 was considered significant. The Kaplan‒Meier method was used to estimate and plot the time curves for the appearance of restenosis in the initial intervention vessels, and the log-rank test was used to compare restenosis between the different sizes of RVD. Statistical analysis was performed using SPSS version 25.0 (*SPSS, Chicago, Illinois*), and figures were plotted by GraphPad Prism software v.8.0 (*GraphPad Software, San Diego, California USA*).

## Results

### Baseline characteristics

**Fig. 1:**
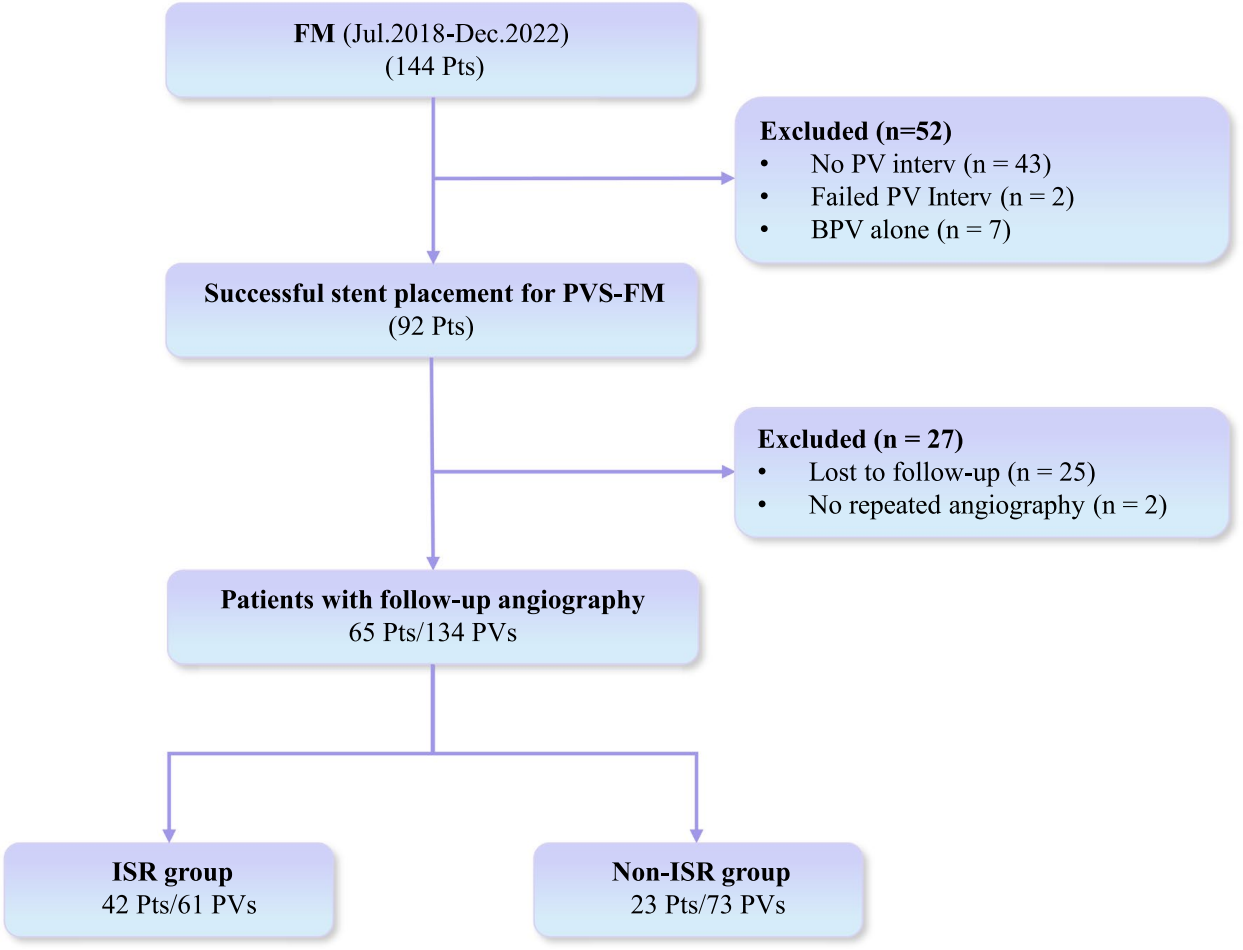
Flowchart of patient enrollment. *BPV* balloon pulmonary venoplasty, *FM* fibrosing mediastinitis, *ISR* in-stent restenosis, *PA* pulmonary artery, *Pts* patients, *PV* pulmonary vein, *PVS* pulmonary vein stenosis

The study flowchart is shown in Fig. 1. Of 144 patients with FM during the study period, 92 patients successfully underwent percutaneous transluminal stent venoplasty. Of them, twenty-five (27.2%) patients were lost to follow-up, 2 (2.2%) had no imaging data at follow-up, and 65 patients (70.7%) with 142 stents implanted in 134 PV lesions during 72 sessions underwent CT and/or selective pulmonary venographic surveillance at a median of 6.6 (3.4–15.7) months of follow-up. Of the 65 patients ultimately included in the analysis, 2 veins were implanted with stents directly without ballooning, and 132 veins were stented after initial balloon angioplasty failed to improve the Pd across the stenotic site. The baseline characteristics are shown in Table 1, and the procedural and lesion characteristics are shown in Table 2.

**Table 1:** Baseline characteristics

	Total (N = 65)	ISR (n = 42)	Non-ISR (n = 23)	*P* Value
*Demographics*				
Age, years	66.0 (60.0, 70.0)	64.5 (59.8, 69.3)	67.0 (60.0, 72.0)	0.38
Body mass index, kg/m^2^ (N = 64)	22.4 ± 3.6	22.2 ± 3.5	22.7 ± 3.7	0.53
Female	29 (44.6)	20 (47.6)	9 (39.1)	0.51
Duration of symptoms, months	37.0 (24.0, 72.0)	38.0 (24.0, 72.0)	36.0 (14.0, 96.0)	0.83
*Clinical presentation*				
Dyspnea	61 (93.8)	38 (90.5)	23 (100.0)	0.29
Cough	20 (30.8)	15 (35.7)	5 (21.7)	0.24
Hemoptysis	4 (6.2)	4 (9.5)	0 (0.0)	0.29
Chest distress	40 (61.5)	22 (52.4)	18 (78.3)	0.040
Palpitations	3 (4.6)	2 (4.8)	1 (4.3)	1.00
Edema of lower limbs	17 (26.2)	12 (28.6)	5 (21.7)	0.55
Pleural effusion	49 (75.4)	32 (76.2)	17 (73.9)	0.84
*Comorbidity*				
Hypertension	21 (32.3)	14 (33.3)	7 (30.4)	0.81
Diabetes mellitus	14 (21.5)	7 (16.7)	7 (30.4)	0.22
COPD	48 (73.8)	29 (69.0)	19 (82.6)	0.23
Atelectasis	42 (64.6)	29 (69.0)	13 (56.5)	0.31
Tuberculosis	39 (60.0)	26 (61.9)	13 (56.5)	0.67
*Hemodynamics (n* = *64)*				
SaO_2_, %	89.0 (87.3, 92.0)	89.0 (87.0, 92.0) (n = 41)	90.0 (88.0, 91.0)	0.83
mRAP, mmHg	3.0 (2.0, 5.0)	3.0 (2.0, 4.5) (n = 41)	5.0 (2.0, 7.0)	0.067
sPAP, mmHg	70.5 (54.0, 80.8)	71.0 (53.5, 81.0) (n = 41)	68.0 (54.0, 78.0)	0.70
dPAP, mmHg	27.0 (22.0, 33.0)	27.0 (22.0, 33.0) (n = 41)	27.0 (20.0, 34.0)	0.80
mPAP, mmHg	39.5 (33.0, 50.0)	40.0 (33.0, 49.5) (n = 41)	39.0 (30.0, 53.0)	0.88
PAWP, mmHg (n = 63)	7.0 (6.0, 9.0)	7.0 (6.0, 9.0) (n = 40)	7.0 (6.0, 7.0)	0.97
PVR, WU	6.3 (5.3, 9.9)	6.3 (5.2, 9.6) (n = 41)	6.8 (5.5, 10.0)	0.48
CO, L/min	4.6 (3.8, 5.4)	4.8 (3.9, 5.5) (n = 41)	4.4 (3.7, 5.3)	0.34
CI, L/min/m^2^	2.8 ± 0.6	3.0 ± 0.7 (n = 41)	2.8 ± 0.6	0.23
SvO_2_, %	62.9 ± 9.0	63.6 ± 9.1 (n = 41)	61.7 ± 9.0	0.52
*Echocardiographic*				
LA size, mm	32.4 ± 5.0	32.4 ± 4.9	32.3 ± 5.2	0.94
TAPSE, mm (n = 50)	19.2 ± 4.6	19.7 ± 4.0 (n = 33)	18.2 ± 5.5 (n = 17)	0.30
RAA, end-systolic, cm^2^ (n = 42)	16.5 (14.0, 20.6)	15.8 (14.0, 22.5) (n = 27)	17.5 (14.3, 20.4) (n = 15)	0.66
RVA, end-diastolic, cm^2^ (n = 42)	24.4 ± 9.2	23.5 ± 9.0 (n = 27)	26.0 ± 9.7 (n = 15)	0.41
*Exercise capacity*				
WHO-FC, I/II/III/IV	0/25/32/8	0/17/19/6	0/8/13/2	0.64
6MWD, m (n = 42)	306.4 ± 97.8	287.6 ± 100.1 (n = 27)	340.2 ± 86.6 (n = 15)	0.16
*Laboratory values*				
NT-proBNP, pg/ml	732.6 (163.5, 1568.5)	714.0 (128.3, 1374.3)	732.6 (203.0, 2678.0)	0.88
NLR (n = 62)	4.7 (3.8, 6.8)	4.6 (3.8, 5.8) (n = 41)	5.2 (3.3, 8.9) (n = 21)	0.48
CRP, mg/L (n = 59)	5.8 (1.7, 14.1)	6.3 (1.7, 16.9) (n = 40)	5.6 (1.7, 9.4) (n = 19)	0.75
D-Dimer, ug/ml (n = 58)	1.0 (0.6, 1.5)	1.0 (0.7, 1.5) (n = 36)	0.9 (0.3, 1.4) (n = 22)	0.22
With PAS	63 (96.9)	41 (97.6)	22 (95.7)	1.00

Values are mean ± SD, n (%), or M (Q1, Q3). *6MWD* 6-min walking distance, *CI* cardiac index, *CO* cardiac output, *COPD* chronic obstructive pulmonary disease, *CRP* C-reactive protein, *dPAP* diastolic pulmonary artery pressure, *ISR* in-stent restenosis, *LA* left atrial, *mPAP* mean pulmonary artery pressure, *NLR* Neutrophil-to-Lymphocyte ratio, *NT-proBNP* N-terminal pro-brain natriuretic peptide, *PAS* pulmonary artery stenosis, *PAWP* pulmonary artery wedge pressure, *PVR* pulmonary vascular resistance, *RA* right atrial, *mRAP* mean right atrial pressure, *RV* right ventricle, *SaO*_*2*_ arterial oxygen saturation, *sPAP* systolic pulmonary artery pressure, *SvO*_*2*_ mixed venous oxygen saturation, *TAPSE* tricuspid annual plane systolic excursion, *WHO-FC* World Health Organization functional class

**Table 2:** Lesion characteristics and procedural-related information

	Total (N = 134)	ISR (n = 61)	Non-ISR (n = 73)	*P* Value
*The lesion distribution, n (%)*				0.21
LSPV	54 (40.3)	29 (47.5)	25 (34.2)	
LIPV	35 (26.1)	14 (23.0)	21 (28.8)	
RSPV	38 (28.4)	17 (27.9)	21 (28.8)	
RIPV	7 (5.2)	1 (1.6)	6 (8.2)	
Cor-PA stenosis	104 (77.6)	54 (88.5)	50 (68.5)	0.006
*Stenosis severity of the Cor-PA*				0.004
Normal	30 (22.4)	7 (11.5)	23 (31.5)	
Mild	36 (26.9)	13 (21.3)	23 (31.5)	
Moderate	23 (17.2)	14 (23.0)	9 (12.3)	
Severe	45 (33.6)	27 (44.3)	18 (24.7)	
MLD, mm	2.2 (1.9, 2.9)	2.1 (1.4, 2.8)	2.4 (2.1, 3.3)	0.003
RVD, mm	7.1 (6.3, 8.6)	7.0 (6.1, 8.2)	7.6 (6.6, 9.6)	0.019
Lesion length, mm (N = 113)	20.1 (15.6, 25.1)	18.5 (15.2, 24.7)	20.8 (16.2, 26.3)	0.35
Diameter stenosis, %	68.1 (60.2, 75.3)	69.7 (62.1, 79.0)	67.1 (59.6, 73.9)	0.080
FLD, mm	6.6 (5.6, 8.1)	6.4 (5.4, 7.4)	7.0 (5.8, 9.0)	0.030
FLD/RVD	0.9 (0.8, 1.0)	0.9 (0.8, 1.0)	0.9 (0.8, 1.0)	0.78
Maximal balloon diameter, mm (N = 132)	7.0 (5.0, 7.0)	6.0 (5.0, 7.0) (n = 59)	7.0 (5.0, 8.0)	0.18
Maximal balloon length, mm (N = 132)	20.0 (20.0, 20.0)	20.0 (20.0, 20.0) (n = 59)	20.0 (20.0, 20.0)	0.94
Maximal balloon pressure, atm (N = 129)	6.0 (6.0, 10.0)	6.0 (6.0, 10.0) (n = 58)	6.0 (6.0, 8.0) (n = 71)	0.13
Balloon-to-vessel ratio (N = 132)	0.8 (0.8, 1.0)	0.9 (0.8, 1.0)	0.8 (0.7, 0.9)	0.006
Stent diameter, mm	7.0 (7.0, 9.0)	7.0 (6.0, 8.0)	7.0 (7.0, 9.0)	0.040
Stent length, mm	19.0 (19.0, 25.0)	19.0 (18.0, 25.0)	20.0 (19.0, 26.0)	0.035
Maximal stent pressure, atm (N = 131)	10.0 (10.0, 12.0)	10.0 (10.0, 12.0) (n = 59)	10.0 (10.0, 12.0) (n = 72)	0.22
Stent-to-vessel ratio	1.0 (1.0, 1.0)	1.0 (1.0, 1.1)	1.0 (1.0, 1.0)	0.023
FLD/Stent	0.9 (0.8, 1.0)	0.9 (0.8, 1.0)	1.0 (0.9, 1.0)	0.069
Overlapping stents, n (%)	8 (6.0)	3 (4.9)	5 (6.8)	0.73
*PVFG, 0/1/2/3 (N* = *133)*				
Preoperative	10/41/82/0	6/21/34/0	4/20/48/0	0.38
Postoperative	0/0/0/133	0/0/0/61	0/0/0/72	1.00
*Pressure gradient, mmHg*				
Pd-pre (N = 126)	24.0 (17.8, 30.0)	24.0 (18.3, 29.8) (n = 56)	25.0 (16.8, 30.0) (n = 70)	0.44
Pd-post (N = 128)	0.0 (0.0, 3.0)	0.0 (0.0, 1.8) (n = 56)	0.0 (0.0, 3.0) (n = 72)	0.34

Values are mean ± SD, n (%), or M (Q1, Q3). *Cor-PA* corresponding pulmonary artery, *DS (%)* percentage diameter stenosis, *FLD* final lumen diameter, *ISR* in-stent restenosis, *LIPV* left inferior pulmonary vein, *LSPV* left supper pulmonary vein, *MLD* minimal lumen diameter, *PA* pulmonary artery, *PVFG* pulmonary venous flow grade, *RIPV* right inferior pulmonary vein, *RSPV* right supper pulmonary vein, *RVD* reference vessel diameter

### Incidence of in-stent restenosis (ISR)

**Fig. 2:**
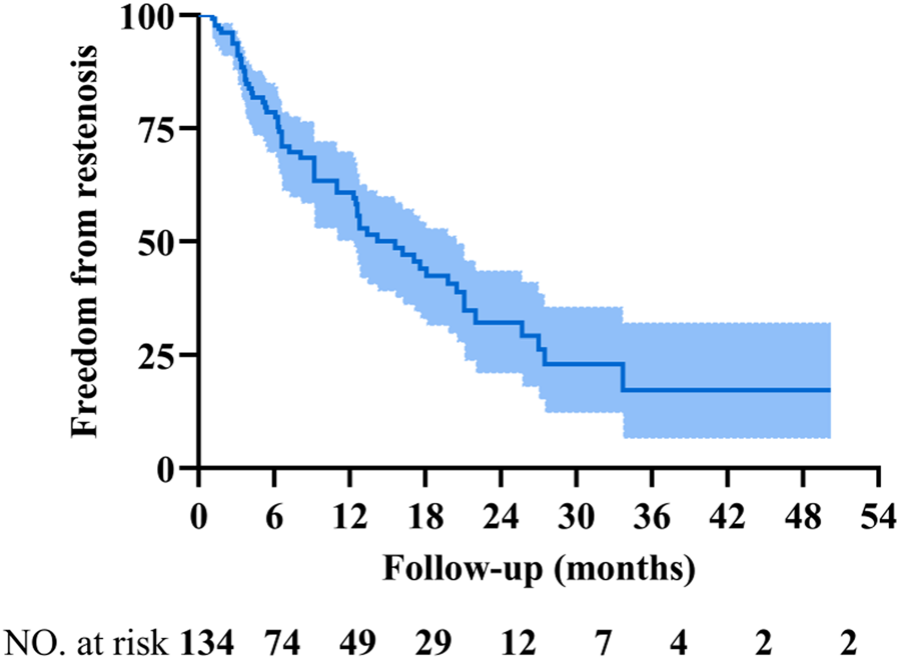
Cumulative incidence of ISR. Kaplan‒Meier curve depicting the probability of ISR over a median of 6.6 (3.4–15.7) months. *ISR* in-stent restenosis

At a median follow-up of 6.6 (3.4–15.7) months, ISR was found in 61 of 134 treated veins. The cumulative ISR was 6.3%, 21.4%, and 39.2% at the 3-, 6-, and 12-month follow-up, respectively (Fig. 2).

### Univariate analysis

Patients with and without ISR had similar ages, sex distribution, body mass index, and medical histories, including chronic obstructive pulmonary disease, diabetes mellitus (DM), tuberculosis, etc. There were also no significant differences in the hemodynamic and laboratory parameters between the two groups. The analysis of clinical factors failed to identify any factors significantly correlated with ISR. Among procedure-related factors, MLD, RVD, FLD, stent diameter, stent length, and stenosis of the corresponding pulmonary artery (Cor-PA) were associated with ISR (Table 3).

**Table 3:** Per-vessel univariate and multivariate analysis associated with in-stent restenosis

	Univariate analysis		Multivariate analysis	
	OR (95% CI)	*P* Value	OR (95% CI)	*P* Value
*The lesion distribution, n (%)*				
LSPV	1 (Ref)			
LIPV	0.58 (0.24–1.36)	0.21		
RSPV	0.70 (0.30–1.61)	0.40		
RIPV	0.14 (0.02–1.28)	0.082		
Cor-PA stenosis	3.55 (1.40–8.99)	0.008	3.41 (1.31–8.86)	0.012
*Stenosis severity of the Cor-PA*				
Normal	1 (Ref)			
Mild	1.86 (0.63–5.50)	0.26		
Moderate	5.11 (1.55–16.81)	0.007		
Severe	4.93 (1.75–13.88)	0.003		
MLD, mm	0.67 (0.48–0.92)	0.013		
RVD, mm	0.78 (0.64–0.96)	0.019	0.79 (0.64–0.98)	0.032
Lesion length, mm	0.98 (0.93–1.03)	0.40		
Diameter stenosis, %	1.02 (0.99–1.05)	0.13		
FLD, mm	0.79 (0.65–0.97)	0.021		
FLD/RVD	0.44 (0.03–7.26)	0.56		
Maximal balloon diameter, mm	0.86 (0.67–1.09)	0.20		
Maximal balloon length, mm	1.00 (0.93–1.09)	0.92		
Maximal balloon pressure, atm	1.14 (0.98–1.34)	0.093		
Balloon-to-vessel ratio	6.62 (0.79–55.82)	0.082		
Stent diameter	0.79 (0.62–0.99)	0.047		
Stent length, mm	0.93 (0.87–0.99)	0.042		
Maximal stent pressure, atm	1.13 (0.96–1.33)	0.14		
FLD/Stent	0.05 (0.00–1.05)	0.053		
Stent-to-vessel ratio	22.62 (0.63–817.65)	0.088		
Overlapping stents, n (%)	0.70 (0.16–3.07)	0.64		
*Pre-PVFG, 0/1/2/3*				
0	1 (Ref)			
1	0.70 (0.17–2.85)	0.62		
2	0.47 (0.12–1.80)	0.27		
*Pressure gradient, mmHg*				
Pd-pre	0.99 (0.96–1.02)	0.53		
Pd-post	0.89 (0.76–1.04)	0.13		

*CI* Confidence interval, *OR* Odds ratio. Abbreviations as in Table 2

### Multivariate analysis

**Fig. 3:**
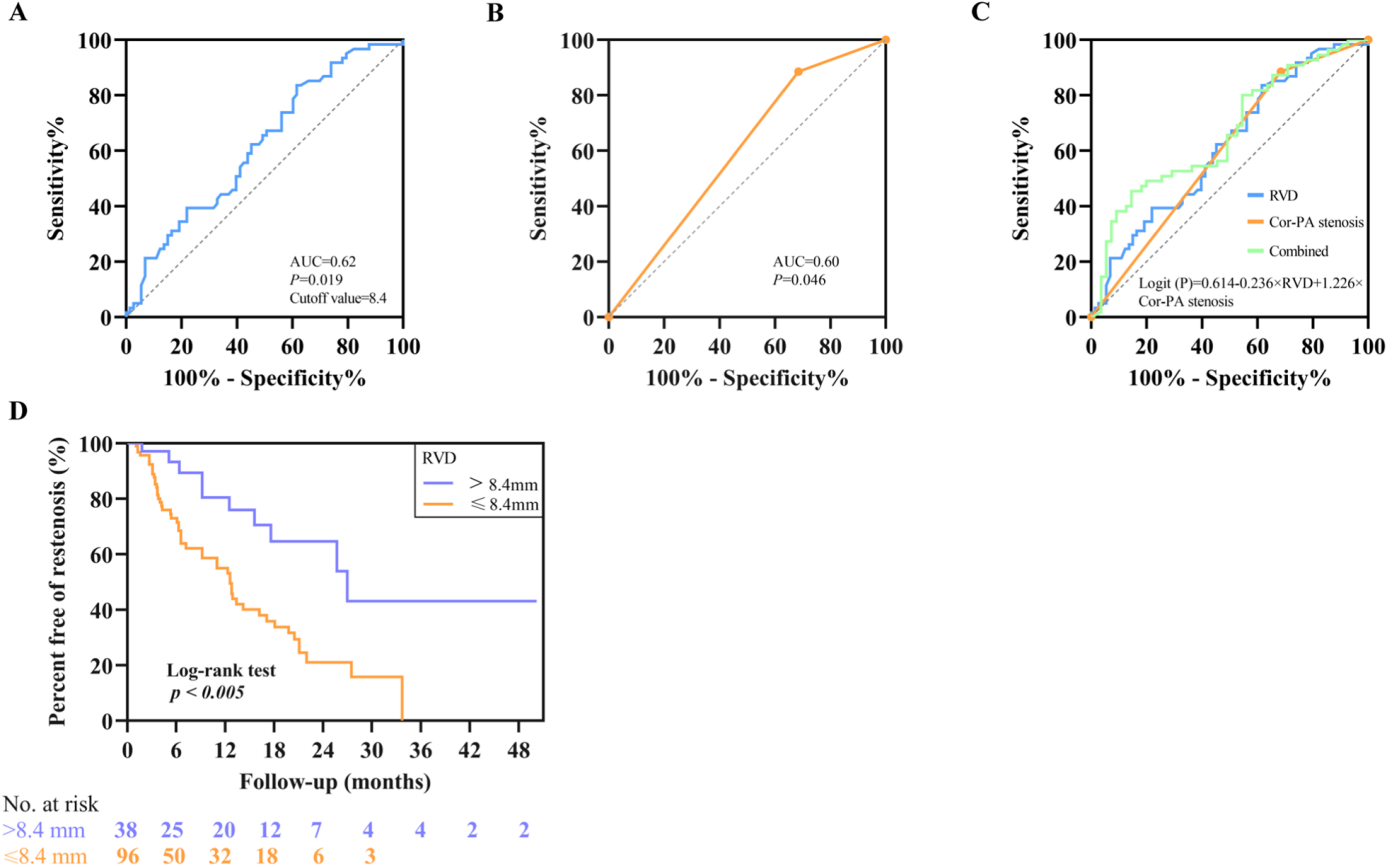
ROC analysis for the determination of ISR in the PVS-FM. (**A**) RVD had a sensitivity and specificity of 0.84 and 0.38, respectively, for a cutoff point of 8.4 mm (AUC, 0.62; 95% CI, 0.52 to 0.71; *P* = 0.019) (blue line). (**B**) Cor-PA stenosis had a sensitivity and specificity of 0.89 and 0.69, respectively (AUC, 0.60; 95% CI, 0.51 to 0.70; *P* = 0.046) (yellow line). (**C**) Binary logistic regression analysis rendered the following formula for the prediction of ISR: Logit (*P*) = 0.614–0.236 × RVD + 1.226 × Cor-PA stenosis (AUC, 0.68; 95% CI, 0.59 to 0.77; *P* < 0.001) (green line). (**D**) Kaplan–Meier survival plot comparing freedom from restenosis stratified by RVD. There was a significant difference (*P* < 0.005 for a log-rank test) between the RVD ≤ 8.4 mm (orange solid line) and > 8.4 mm (purple dashed line) groups. *AUC* area under the curve, *CI* confidence interval, *Cor-PA* corresponding pulmonary artery, *ISR* in-stent restenosis, *PVS-FM* pulmonary vein stenosis caused by fibrosing mediastinitis, *RVD* reference vessel diameter

To ensure no multicollinearity among the variables, the appropriate variables were selected by calculating the tolerance and variance inflation factor (VIF). Then, multivariate analysis was conducted on the remaining variables. Procedure-related parameters independently associated with ISR included the RVD and Cor-PA stenosis **(Central illustration)**. For ISR, RVD was associated with an adjusted OR of 0.79 (95% CI, 0.64 to 0.98,* P* = 0.032), while the stenosis of Cor-PA was associated with an adjusted OR of 3.41 (95% CI, 1.31 to 8.86, *P* = 0.012). The results of the ROC analysis for procedure-related variables for ISR are depicted in Fig. 3. RVD > 8.4 mm could be used as the cutoff point to predict ISR, and its sensitivity and specificity were 0.84 and 0.38, respectively. The subgroup of vessels with a reference diameter > 8.4 mm had a significantly lower risk of ISR than the subgroup with a reference diameter ≤ 8.4 mm **(**Fig. 3D**)**. Meanwhile, the sensitivity and specificity of Cor-PA stenosis were 0.89 and 0.69, respectively. When the positive and negative influencing factors were combined, the sensitivity and specificity were 0.77 and 0.55, respectively. Hence, we obtained the regression equation of related variables and restenosis: Logit (*P*) = 0.614–0.236 × RVD + 1.226 × Cor-PA stenosis (Cor-PA stenosis = 1 if present, 0 absent).

**Central illustration** Based on the constructed prediction model, the RVD and stenosis of Cor-PA were found to be independently associated with ISR, and their sensitivity and optimal cutoff values for the prediction of restenosis are shown in (a) and (b), respectively. The risk of ISR significantly increased when PA stenosis occurred; the risk of restenosis decreased significantly when the RVD was larger than 8.4 mm. Cor-PA corresponding pulmonary artery, ISR in-stent restenosis, PA pulmonary artery, RVD reference vessel diameter. 

### Procedural complications

In the analysis of 72 sessions performed in 65 patients, there were 13 episodes of chest tightness (18%) and 14 episodes of cough (19%), which were the most common during the intervention **(**Supplemental Table 1**)**. Mild hemoptysis and transient cardiac arrest/bradycardia occurred in 7 and 4% of sessions, respectively, with no requirement for additional intervention. There were 2 (3%) patients experiencing PV dissection/perforation who underwent balloon occlusion with low inflation pressure and recovered without any hemodynamic insults. One patient suffered from suspected acute pulmonary edema with acute onset of dyspnea and elevated left atrial pressure after stent implantation, high flow oxygen, and diuretics were administered, and these symptoms were relieved soon after. There were no cases of peri-procedure death or major hemoptysis occurred.

### Immediate and short-term efficacy

**Fig. 4:**
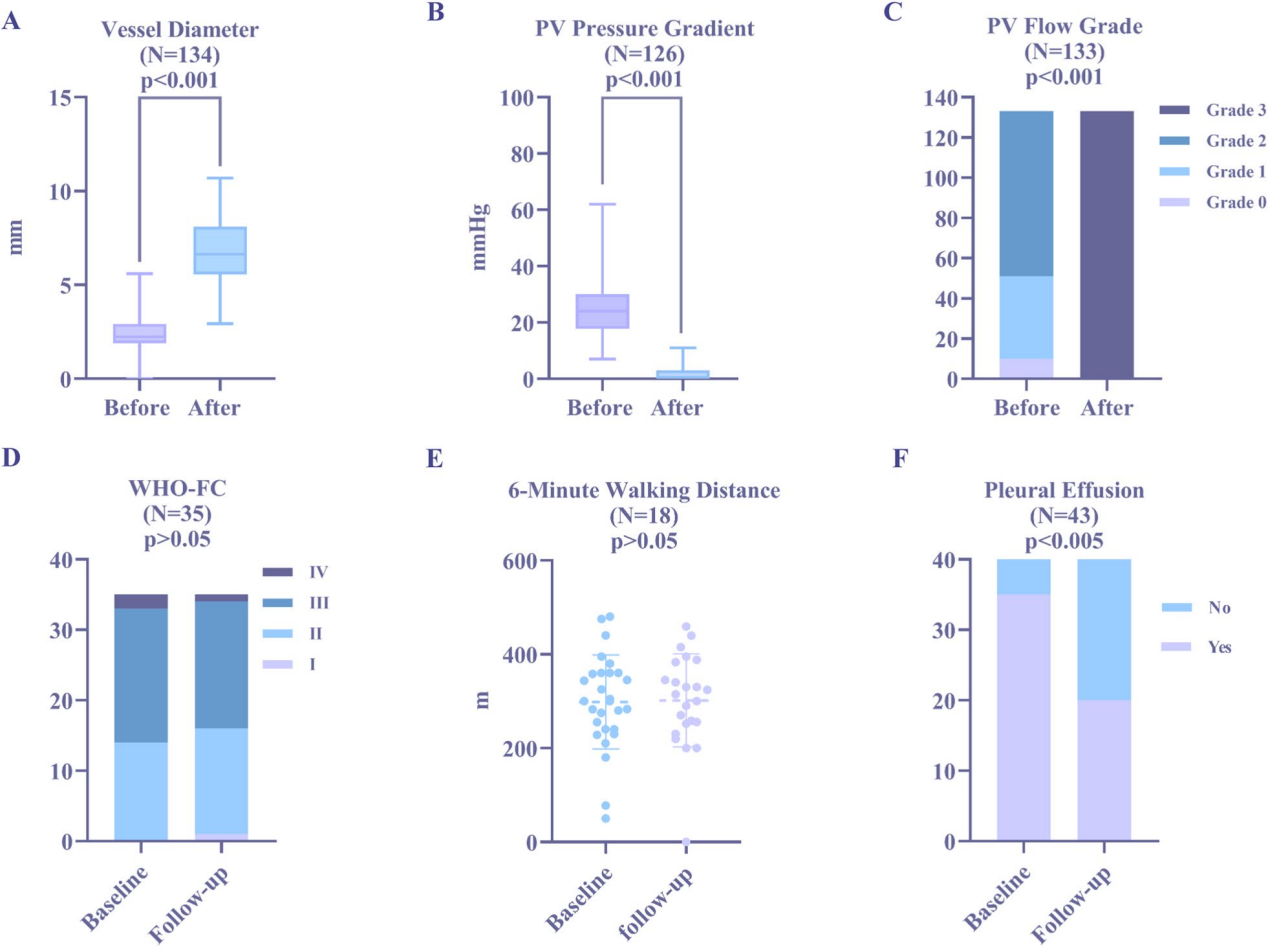
Immediate and short-term efficacy. **A-C** show the immediate effects of the intervention. **D-F** show the short-term effects of PV intervention alone. When the pre- and postintervention data were compared, there was a significant improvement in MLD, Pd, and PVFG (*P* < 0.001 for all). There was a significant improvement in pleural effusion but no changes in WHO-FC and 6MWD after PV intervention compared with baseline (*P* < 0.005, *P* > 0.05, and *P* > 0.05, respectively). *6MWD* 6-min walking distance, *MLD* minimal lumen diameter, *Pd* pressure gradient, *PV* pulmonary vein, *PVFG* pulmonary venous flow grade, *WHO-FC* World Health Organization functional class

The MLD, Pd, and PVFG of the recruited patients were evaluated pre- and post-intervention and drastically improved after the intervention. The MLD increased from 2.2 (1.9, 2.9) mm to 6.6 (5.6, 8.1) mm, and PVFG and Pd were significantly improved (*P* < 0.001 for all)** (**Fig. 4A–C**)**. Additionally, due to 17 patients undergoing PA intervention at the same time or later, the short-term efficacy of the remaining 43 (66.2%) patients with only PV intervention at 5.0 (3.1–11.2) months follow-up was analyzed. Among the 43 patients, 23 underwent right heart catheterization during the follow-up. Comparisons of the baseline and follow-up data in patients with PV intervention are shown in Table 4. The pleural effusion decreased from 35 (81.3%) to 20 (46.5%) (3 of which were new pleural effusions) (*P* < 0.005) during the follow-up. However, there was no significant improvement in the postoperative WHO-FC or 6MWD (*P* > 0.05)** (**Fig. 4D–F**)**. The mean PAP had a significant improvement (*P* = 0.016), and there was an increase in left atrial size (*P* = 0.44) but with no statistical significance.

**Table 4:** Short-term efficacy of percutaneous pulmonary venoplasty in patients with PVS-FM

	Baseline	Follow up	N	*P* Value
*Exercise capacity*				
6MWD, m	307.6 ± 107.1	326.1 ± 78.2	18	0.28
WHO-FC, I/II/III/IV	0/14/19/2	1/15/18/1	35	0.49
*Hemodynamics*				
sPAP, mmHg	69.3 ± 22.8	58.9 ± 19.0	23	0.024
dPAP, mmHg	27.0 (23.0, 36.0)	27.0 (17.0, 33.0)	23	0.008
mPAP, mmHg	39.0 (33.0, 52.0)	34.0 (30.0, 44.0)	23	0.016
PAWP, mmHg	7.0 (6.0, 9.3)	8.0 (4.0, 11.0)	23	0.39
mRAP, mmHg	3.0 (2.0, 6.0)	3.0 (2.0, 4.0)	23	0.54
PVR, WU	6.9 (5.3, 10.0)	6.3 (3.9, 9.5)	23	0.32
SvO_2_, %	62.0 (57.0, 68.0)	66.0 (58.0, 70.0)	23	0.69
CO, L/min	4.3 ± 1.3	4.4 ± 1.2	23	0.69
CI, L/min/m^2^	2.7 ± 0.7	2.8 ± 0.7	23	0.57
*Echocardiographic*				
LA size, mm	32.2 ± 5.4	32.8 ± 3.8	39	0.44
TAPSE, mm	17.3 ± 4.5	19.5 ± 3.9	30	0.016
RAA, end-systolic, cm^2^	16.2 (14.1, 20.3)	17.0 (14.0, 21.8)	21	0.73
RVA, end-diastolic, cm^2^	25.1 (16.3, 34.9)	28.5 (19.9, 37.7)	20	0.018
*Others*				
SaO_2_, %	89.3 ± 4.9	88.1 ± 5.9	38	0.38
NT-proBNP, pg/ml	738.0 (203.0, 2678.0)	360.1 (145.3, 1602.5)	41	0.74
Refractory pleural effusion, n (%)	35 (81.3)	20 (46.5)	43	< 0.005
CRP, mg/L	6.3 (2.5, 29.5)	4.0 (1.4, 16.1)	37	0.054
D-Dimer, ug/ml	1.2 ± 0.7	1.3 ± 1.0	36	0.67

Abbreviations as in Table 1

## Discussion

In this study, we focused on the incidence and predictors of ISR in PVS-FM. The salient findings are as follows: (1) the incidence of ISR following stent implantation of PVS-FM is as high as 6.3, 21.4, and 39.2% at 3-, 6-, and 12-month follow-ups, respectively. (2) RVD is an independent factor for ISR, and the stenosis of the Cor-PA largely affects the occurrence of restenosis.

### The rate of in-stent restenosis (ISR) and its associated factors

Previously, Albers et al. [5] reported a restenosis rate of 7/16 (44%) patients with PVS-FM during a median 115-month follow-up. Similarly, the Mayo Clinic experience described a restenosis rate of up to 4/8 (50%) in PVS-FM patients after the intervention [4]. However, the sample size of the above studies was small. In our study, CT angiography was routinely performed to identify ISR in 134 PVs of 65 patients with PVS-FM. A total of 61/134 (45.5%) PVs and 42/65 (64.6%) patients had ISR during a median of 6.6 months of follow-up. The cumulative ISR was 6.3, 21.4, and 39.2% at the 3-, 6-, and 12-month follow-ups. Accordingly, this study confirms the high ISR rate with more detailed and accurate information in a larger cohort of PVS-FM patients. A high restenosis rate was also reported in PVS with other etiologies. In PVS-PVI, the restenosis rate is between 19 and 39% after a median follow-up period of 6.0–55.2 months [17–21]. Hence, ISR in PVS-FM could be higher than that in PVS-PVI. The explanation for the higher ISR in PVS-FM than in PVI-PVS is as follows. (1) The involved PVs are often different in diameter. PVI-PVS after PVI is always located at the ostia of PV with a larger caliber, while PVS-FM is at the proximal 1st tributary of PV with a smaller caliber; and (2) different pathogenesis could be another attribute. PVS-PVI is intraluminal intimal hyperplasia caused by thermal injury, while PVS-FM is extraluminal due to proliferative fibrous tissue compression. Also, PVS-FM might be more easily injured by balloon or stent inflation than PVS-PVI. (3) Accompanied PA stenosis is common in PVS-FM, which could be a unique feature in FM in China [2, 22].

Earlier studies showed some factors of restenosis after percutaneous coronary intervention, including vessel size, maximal balloon pressure, stent type, and final diameter stenosis [23]. In this study, the predictors of ISR were analyzed using a multivariable logistic model. We found that the RVD and Cor-PA stenosis were independent predictors of ISR following PV intervention in FM. Previous research has established a correlation between stent size and the risk of restenosis in PVS-PVI. Prieto et al. [21] reported that a stent diameter smaller than 10 mm may increase the risk of restenosis in PVS-PVI patients. Subsequently, this finding was corroborated in pediatric PVS by Balasubramanian et al. [24], who observed that a stent size ≥ 7 mm was associated with lower restenosis. Hence, some scholars have advised that a stent diameter exceeding 8 mm could be a preferred initial choice for PV interventions [17]. Additionally, a mismatch between the stent and the vessel might increase the occurrence of restenosis [25]. Our findings are consistent with previous investigations to some extent. And the result is more rational because the choice of stent size is based on the RVD.

Previously, some studies showed that stent angioplasty results in less restenosis than balloon dilation after a successful PV intervention [18, 21]. Drug-eluting stents have been widely used in coronary artery disease to prevent restenosis [25]. In contrast, Fink et al. [20] reported a high incidence of restenosis after treatment with a drug-eluting stent and a favorable outcome following intervention with large-diameter bare metal stent implantation, which emphasizes the importance of choosing a stent of the right size. Recently, a meta-analysis depicted that the overall restenosis rate was 54% in BA and 22.3% in stenting of PVS with different etiologies, during a median follow-up time of 13–69 months [26]. Either over- or underinflation of the balloon or stent may affect restenosis, which has been demonstrated in cases of coronary artery intervention [27–29]. In a previously published investigation, Masaki and his team revealed that rapamycin-eluting films could suppress the progression of pulmonary vein obstruction [30], which is promising for ISR in PV intervention. The latest case report from the United States confirmed that the use of drug-coated balloons revealed no evidence of restenosis [31]. In other words, further study is necessary in the future.

As expected, Cor-PA stenosis is an exclusive factor associated with ISR in FM, which should be given more attention in PV intervention. Wang et al. [2] previously classified FM into 3 types: only the artery involved, only the vein is compressed, and there is both artery and vein narrowing, which should be a mandatory evaluation for an interventional strategy of patients with FM. Overall, the ISR following PV intervention is significantly higher than that following PA intervention, regardless of the etiology of PVS, which may be attributed to the lower pressure in the venous system [20]. In conclusion, paying attention to the importance of Cor-PA stenosis is critical.

### Clinical significance and roles of RVD and cor-PA stenosis

In our study, the RVD served as an independent predictor of ISR, with larger RVDs indicating a reduced risk of ISR, and early referral may allow for timely interventions that minimize reference vessel atrophy, thereby improving long-term patency [21]. Previous study also confirmed our finding that small RVD had higher restenosis [21]. Therefore, smaller RVD may require more complex interventions to prevent ISR. In summary, we underscore the importance of great caution in the treatment of smaller PVs, particularly given the prevalent stenosis observed in these vessels among patients with FM. This poses a formidable challenge to the effective management of FM, thereby necessitating more research to provide novel therapeutic strategies. Furthermore, we emphasize the avoidance of undersized stent implantation, as it is associated with an elevated risk of restenosis, thereby emphasizing the importance of precise stent sizing and selection in interventional procedures.

The narrowing of the Cor-PA has emerged as a pivotal factor that substantially elevates the risk of ISR. This phenomenon is primarily attributed to the alteration in hemodynamic states and the intensified complexity of interventional procedures. Consequently, the meticulous quantification of the stenosis severity, coupled with a thorough assessment of its anatomical location during the preoperative period, holds paramount importance in tailoring and executing individualized therapeutic strategies. The significance of this finding lies in several aspects: (1) To emphasizing the importance of preoperative assessment, which involves the simultaneous evaluation of both PAs and veins, as well as the crucial of clinical subtypes. (2) For Type III (referring to the FM eliciting stenosis of the PAs, PVs, and bronchi), it is crucial to simultaneously restore patency to both PVs and PAs, or promptly restore patency to the PA after the narrowing PV has regained patency. (3) In cases where the PA occlusion is unlikely to be restored, further intervention on the PVs is not recommended [2]. This decision-making process is grounded in profound pathophysiological insights and the accumulated wisdom of clinical practice.

In summary, RVD and Cor-PA stenosis emerge as significant predictors of ISR, demonstrating remarkable clinical value in guiding the planning and refinement of treatment protocols. By systematically integrating and analyzing these crucial factors, we can more precisely select treatment modalities, aiming to reduce ISR risk and improve patient outcomes.

### The safety and efficacy of PV intervention

A previous study in 8 patients with PVS-FM demonstrated that the incidence of peri-procedure complications and mortality is as high as 3/8 (37.5%) and 3/8 (37.5%), respectively[4]. Another study showed that overall procedure-related complications in patients with FM, including PA, PV, and SVC intervention, were 15/58 (26%) minor and 6/58 (10%) severe [5]. In the present large cohort study, we found that some patients had a cough (19%), chest tightness (18%), and other discomfort (18%), including palpitations, dizziness, nausea, etc. No major hemoptysis or periprocedural death occurred. The incidence and severity of complications in this study are different from the previous description, which could be attributed to concomitant conditions, inflation pressure, location of PV lesion, patient status, and proficient techniques. There is a compelling indication for the early referral of patients with FM to specialized centers, equipped with substantial expertise in interventional therapies.

In this study, there were immediate improvements in PV caliber, Pd across lesions, and PVFG after the intervention compared with before the intervention, which further supported the findings in previous small sample-sized studies [4, 5]. The PAP evaluated by right heart catheterization decreased in the follow-up period. Notably, the lack of improvement in exercise capacity (6MWD, WHO-FC), despite showing a trend of improvement, may be attributed to the fact that some of the patients experienced a recurrence of symptoms and required further intervention (nearly 50%) during follow-up. On the other hand, remaining PA stenosis may negatively influence the overall efficacy. Hence, further long-term follow-up is needed to analyze the efficacy of PV intervention with the PVS-FM.

## Limitations

The present study is subject to several limitations. Firstly, the assessment of the degree of ISRs relied on CT angiography, which may potentially underestimate or overestimate the extent of restenosis. Secondly, this study was a retrospective study with a small sample size and single-center data. However, ours is one of the largest patient series to date. Thirdly, the incompleteness of follow-up data is noteworthy, as a proportion of patients were lost to follow-up. Lastly, no long-term efficacy was followed up because a substantial number of patients underwent subsequent PA intervention.

## Conclusions

The ISR is very high after the initial intervention of PVS-FM, which is independently associated with RVD and the stenosis of Cor-PA.

## Supplementary Information

Additional file 1.

## Data Availability

Anonymized data used and/or analyzed during the current study are available from the corresponding author upon reasonable request from any qualified investigator for the sole purpose of the study.

## Abbreviations

Cor-PA: Corresponding pulmonary artery
FLD: Final lumen diameter
FM: Fibrosing mediastinitis
ISR: In-stent restenosis
LA: Left atrium
MLD: Minimal lumen diameter
PA: Pulmonary artery
Pd: Pressure gradient
PTPV: Percutaneous transluminal pulmonary venoplasty
PV: Pulmonary vein
PVFG: Pulmonary venous flow grade
PVI: Pulmonary vein isolation
PVS: Pulmonary vein stenosis
PVS-FM: Pulmonary vein stenosis caused by fibrosing mediastinitis
RVD: Reference vessel diameter
