# Supplementary material for: Incidence and predictors of in-stent restenosis following intervention for pulmonary vein stenosis due to fibrosing mediastinitis
Source: Orphanet J Rare Dis. 2024 Oct 14;19:379. doi: 10.1186/s13023-024-03391-8 (PMC11472477; doi:10.1186/s13023-024-03391-8)
Supplement: Supplementary file 1 — Additional file 1. [file 13023_2024_3391_MOESM1_ESM.docx]

**Supplemental Table 1 Procedure-related complications during the session**

| **Complications** | **N=72** |
| --- | --- |
| Cough | 14 (19) |
| Shortness of breath | 7 (10) |
| Chest tightness | 13 (18) |
| Chest pain | 5 (7) |
| Mild hemoptysis | 5 (7) |
| PV dissection/perforation | 2 (3) |
| Transient right bundle branch block | 0 (0) |
| Transient sinus arrest/bradycardia | 3 (4) |
| Embolic stroke | 0 (0) |
| Pericardial tamponade | 0 (0) |
| PV rupture | 0 (0) |
| Pulmonary edema | 1 (1) |
| Other symptoms^*^ | 13 (18) |

Data presented as n (%).

^*^Including palpitations, dizziness, nausea, low blood pressure, tightness of the neck, sweating, etc. *N* number of sessions.
